# Evaluation of interactive digital art healing effects based on multi modal physiological signals and generative adversarial networks (GANs): an empirical study of the neural mechanisms of emotion regulation

**DOI:** 10.3389/fpsyg.2026.1832572

**Published:** 2026-05-22

**Authors:** Zan Xu, Bo Xia

**Affiliations:** School of Architecture and Art, Taiyuan University of Technology, Jinzhong, China

**Keywords:** digital art therapy, emotional regulation, generate adversarial networks, neural mechanism, perceived social support

## Abstract

**Introduction:**

With the global mental health crisis intensifying, traditional psychological interventions face challenges of resource inequity and limited cultural adaptability. Digital technology offers innovative pathways for emotional regulation. This study investigates a digital art therapy system integrating generative adversarial networks (GANs) with multimodal physiological signals to uncover its mechanisms for influencing emotional regulation through neural and social-behavioral pathways.

**Methods:**

Through controlled laboratory experiments and a mixed-method design, this study systematically examined how GAN-generated content types directly affect emotional regulation. A total of 120 adult participants were recruited and randomly assigned to static, dynamic, and interactive intervention groups. The mediating roles of perceived social support and technology acceptance, as well as the moderating effects of cultural capital and social network density, were further analyzed.

**Results:**

Interactive digital content significantly enhanced emotional stability and social adaptability by strengthening user autonomy and virtual community engagement. This effect operated through a chain-mediation pathway involving perceived social support and technology acceptance. Individuals with high cultural capital demonstrated greater emotional improvement, while dense social networks amplified the intervention effects by accelerating support resource transmission.

**Discussion:**

This study proposes a triadic interaction model for digital art therapy, extending traditional theoretical frameworks and offering a new perspective on human-computer collaborative emotional regulation. The findings provide empirical evidence for optimizing interactive design, enhancing cultural adaptation, and improving fairness in technological solutions, thereby advancing digital mental health services toward greater precision and inclusivity.

## 1 Introduction

Contemporary society is facing increasingly severe mental health challenges, and the global prevalence of emotional disorders such as anxiety and depression has become an undeniable public health issue. Traditional psychological intervention methods are limited by uneven distribution of resources, social stigmatization, and low individual participation willingness, making it difficult to meet the needs of diverse groups (Zhao et al., 2024; Xi and Wu, 2024). In this context, the rapid development of digital technology has opened up a new path for the field of mental health, especially the integration of generative adversarial networks and multi modal physiological signal technology, providing precise and personalized solutions for emotional regulation. Digital art therapy, as an emerging form of intervention, reconstructs the interaction between individuals and technology through immersive interactive experiences. Its core value lies in the organic combination of aesthetic expression, neuroscience principles, and social behavior mechanisms, thereby breaking through the temporal and cultural barriers of traditional healing models (Xia et al., 2024). This technological paradigm not only responds to the urgent demand for mental health services in society, but also redefines the role boundaries of human-machine collaboration in emotional support systems.

The evaluation of the effectiveness of digital art therapy needs to be based on an interdisciplinary perspective and a deep analysis of its underlying mechanisms. Existing research mostly focuses on single dimensional verification of technological implementation or clinical efficacy, lacking systematic exploration of the complex interaction chain of “technology neural society” (Wen, 2025). Specifically, how does the virtual environment constructed by generative adversarial networks affect emotional regulation through neural plasticity? How does social and cultural capital shape individuals' acceptance and adaptability to technological interventions? The answers to these questions require researchers to go beyond the limitations of technological instrumentalism and instead focus on the dynamic balance of cognitive, emotional, and social factors in human-machine symbiotic relationships (Xie et al., 2025; Wu et al., 2025). This study attempts to construct a theoretical framework that integrates neural mechanisms and social behavioral variables, revealing the multi-level pathways of digital art therapy in emotional regulation, and providing a more explanatory scientific basis for technology enabled mental health interventions.

From the perspective of wider practical applications, the value of digital art healing lies not only in proposing a new technical model, but also in providing an extensible auxiliary path for the current mental health support system with tight resources, insufficient service accessibility and obvious cultural differences. Compared with the traditional intervention methods that rely heavily on offline professionals and fixed fields, the moderately interactive digital content is easier to be embedded in scenes such as college psychological support, community emotional service, hospital rehabilitation company and online public health platform, providing more convenient front-end support for people with mild to moderate emotional distress. This paper focuses on perceived social support, technology acceptance, cultural capital and social network density, not to increase the complexity of the model, but to answer a more realistic question, that is, why the same digital intervention will produce differentiated effects in different populations. Only by combining the technical design with the social resource conditions of users, can digital healing really move from proof of concept to a more scalable, adaptable and inclusive public application practice.

## 2 Theoretical basis

### 2.1 Social function theory of art healing

Digital art therapy, as an emerging social practice, revolves around reconstructing the interaction between individuals and communities through creative expression (M2 Presswire, 2025). Traditional art therapy emphasizes the repairing effect of manual creation on psychological trauma, while the intervention of digital technology breaks through the limitations of physical media, shifting artistic expression from static works to dynamic interactive experiences (Mersal et al., 2025; Qiu et al., 2025; Ren, 2025). The immersive environment created by technologies such as virtual reality and generative adversarial networks not only provides a safe space for emotional release, but also stimulates users' desire for self exploration through personalized content generated by algorithms (Li, 2025). This technology empowered healing model is essentially an extension of Bourdieu's theory of cultural capital—digital literacy has become a new form of cultural capital that determines an individual's emotional regulation ability in the technological society (Wang et al., 2024; Xian, 2024). At the same time, the rise of online art communities indicates that digital art therapy is reshaping the paradigm of collective emotional support, enabling dispersed individuals to form emotional resonance networks through virtual collaboration (Ma and Ma, 2024; Jiang and Chen, 2023; Langford, 2019).

### 2.2 Social behavior research on multi modal physiological signals

Physiological signals, as objective representations of emotional states, provide a bio social analysis pathway for understanding social behavior (Jang et al., 2025a,b). The lateralization of alpha waves in EEG signals reveals the neural basis of emotional valence, while heart rate variability maps the dynamic response of the autonomic nervous system to social stress. These physiological indicators not only quantify the effect of emotional regulation, but more importantly, they reveal how cultural norms shape individuals' biological response patterns (Ebato et al., 2025; Zhang et al., 2025; Yang et al., 2025). For example, individuals in collectivist cultures may exhibit stronger tendencies toward emotional suppression, reflected in differences in baseline levels of skin conductance response (Jang et al., 2025a,b). The interaction between physiological signals and cultural backgrounds suggests that the design of digital therapy technology needs to balance biological universality and cultural specificity, avoiding imposing Western centered emotional expression paradigms on diverse groups (Polo et al., 2025).

### 2.3 Social application controversy in generative adversarial networks

**Figure 1:**
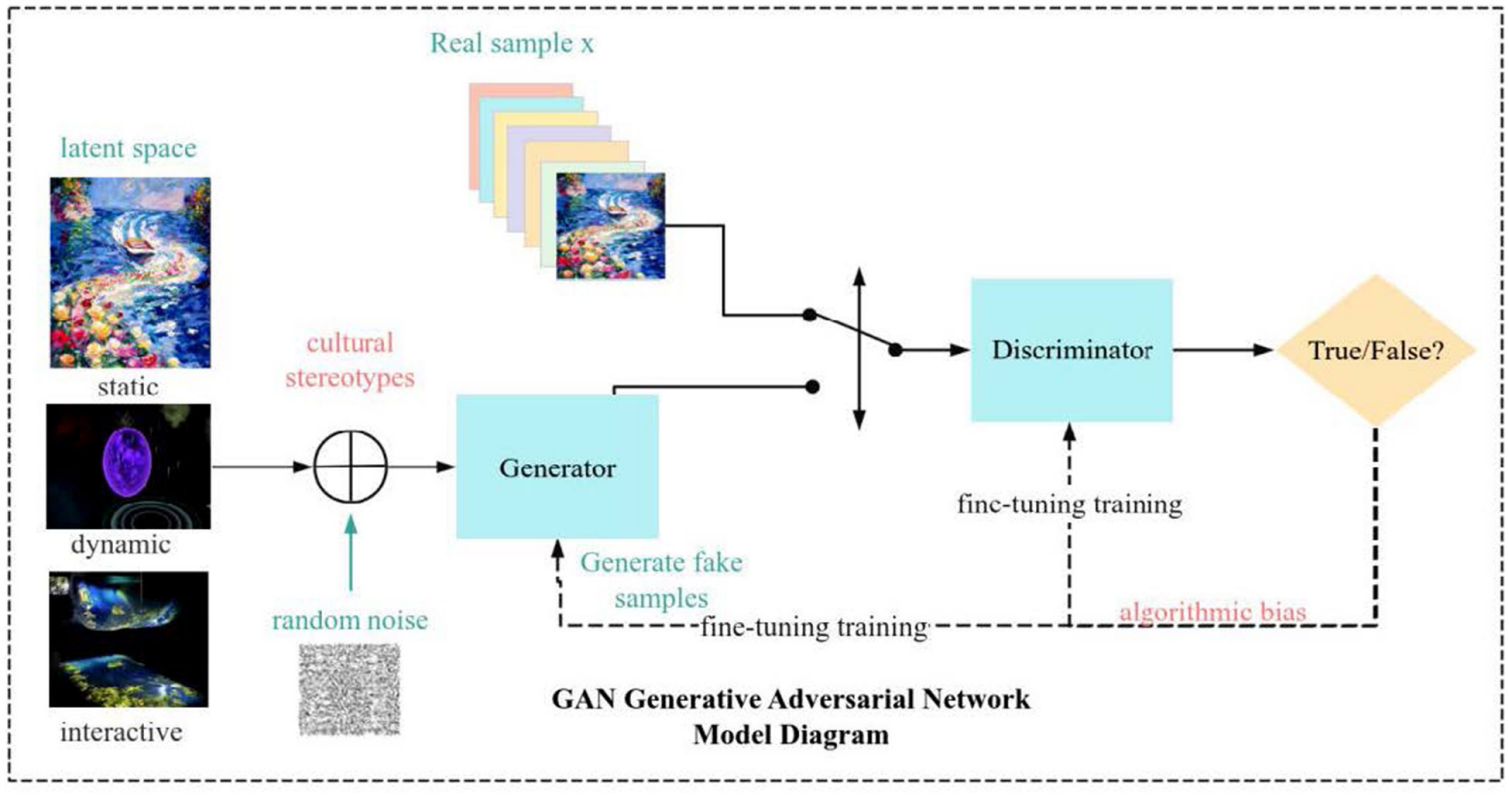
Adversarial network model for healing content generation.

The application of generative adversarial networks in artistic creation has sparked a deep discussion on the authenticity of technology and cultural sovereignty (Figure 1). On the one hand, GAN expands the boundaries of artistic expression through style transfer and content generation, enabling non-professional groups to easily participate in creation, which is seen as a progress in technological democratization (Singh et al., 2025). On the other hand, the algorithm generated “synthetic aesthetics” may dissolve the cultural context of original art, leading to the flattening of symbolic meanings. For example, random collages of ethnic clothing elements may exacerbate cultural stereotypes, while algorithmic bias may systematically marginalize the aesthetic expression of minority groups (Wang et al., 2025; Alejandro et al., 2025). The contradiction between technological empowerment and symbolic alienation requires researchers to establish ethical review mechanisms when developing healing systems, ensuring that the generated content respects cultural diversity while maintaining individual narrative sovereignty (Peng et al., 2025).

### 2.4 Neurosocial interaction theory of emotion regulation

The research on the neural mechanism of emotion regulation reveals the top-down regulation path of the prefrontal cortex to the limbic system, and the intervention of social science further expands the explanatory dimension of this model (Li et al., 2025; Tian et al., 2025). Social support networks can enhance individuals' cognitive reappraisal ability toward negative emotions by strengthening the functional connectivity of the dorsolateral prefrontal cortex. This neuroplasticity phenomenon confirms the shaping effect of social environment on brain structure (Gao et al., 2025). Meanwhile, Hochschild's theory of emotional labor suggests that social role expectations force individuals to adopt specific regulatory strategies, and this long-term behavioral pattern may affect the functional integration of default pattern networks through neural remodeling (Barnett and Vasiu, 2024). The dialogue between neuroscience and social theory suggests that emotional regulation is both an adaptive response driven by biological instincts and a result of internalizing social norms. This dual attribute provides a theoretical fulcrum for constructing interdisciplinary models of technological interventions (Lang et al., 2024; Li et al., 2024).

### 2.5 Research hypothesis

H1: GAN content type affects emotional regulation through a chain mediated effect of social support perception and technology acceptance.

Based on the explanatory framework of social support theory for the effectiveness of technology intervention, previous studies have found that individuals' perceived social support in technology interaction can enhance their trust in the usefulness of technology, thereby promoting the active use of emotion regulation strategies (Lin, 2025). In digital art healing scenarios, the virtual environment constructed by generative adversarial networks may enhance users' sense of identification and willingness to participate in technological interventions by simulating social support scenarios. Therefore, this study proposes hypothesis.

**Figure 2:**
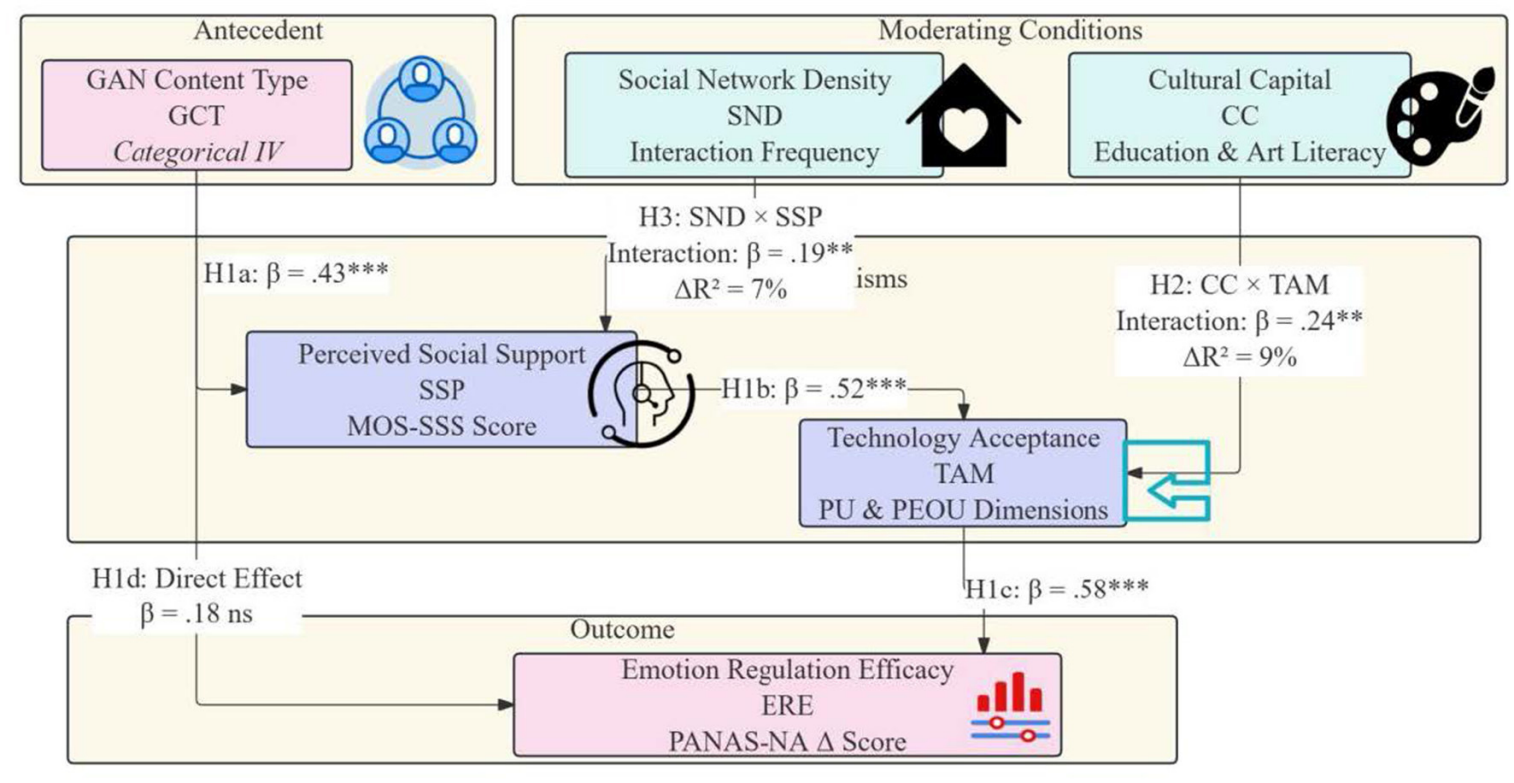
Research hypothesis path diagram. ^**^p < 0.01; ^***^p < 0.001.

H1: generative adversarial network content types have a significant mediating effect on emotional regulation through the chain of social support perception and technology acceptance, and dynamic and interactive content will exhibit stronger mediating effects (Figure 2).

H2: the relationship between cultural capital positively regulating technological acceptance and emotional regulation effects.

Bourdieu's theory of cultural capital reveals the shaping effect of social resource allocation on individual technological adaptability. Previous studies have shown that high cultural capital groups are more likely to understand the symbolic value of technological products and transform it into behavioral advantages for emotional regulation (Xie and Hutagalung, 2025). Education level and artistic literacy, as core dimensions of cultural capital, may enhance users' cognitive decoding ability of technological intervention logic, thereby amplifying the promoting effect of technological acceptance on emotional improvement. Based on this, this study proposes hypothesis.

H2: cultural capital positively moderates the relationship between technology acceptance and emotional regulation effects, with high cultural capital groups having significantly higher regulatory effects than low cultural capital groups.

H3: social network density positively moderates the impact of perceived social support on emotional regulation.

Social network theory emphasizes the reinforcement mechanism of group interaction on individual behavioral decision-making. Empirical research has shown that high-density social networks can accelerate the efficiency of social support transmission through information sharing and emotional resonance (Zheng and Sikora, 2025). In the process of digital art therapy, the density of virtual communities embedded by users may determine their ability to access social support resources, thereby affecting the emotional regulation effectiveness of technological interventions. Therefore, this study proposes hypothesis H3: social network density positively moderates the impact of perceived social support on emotional regulation, and the emotional improvement effect of perceived social support is more significant in high-density network environments.

**Figure 3:**
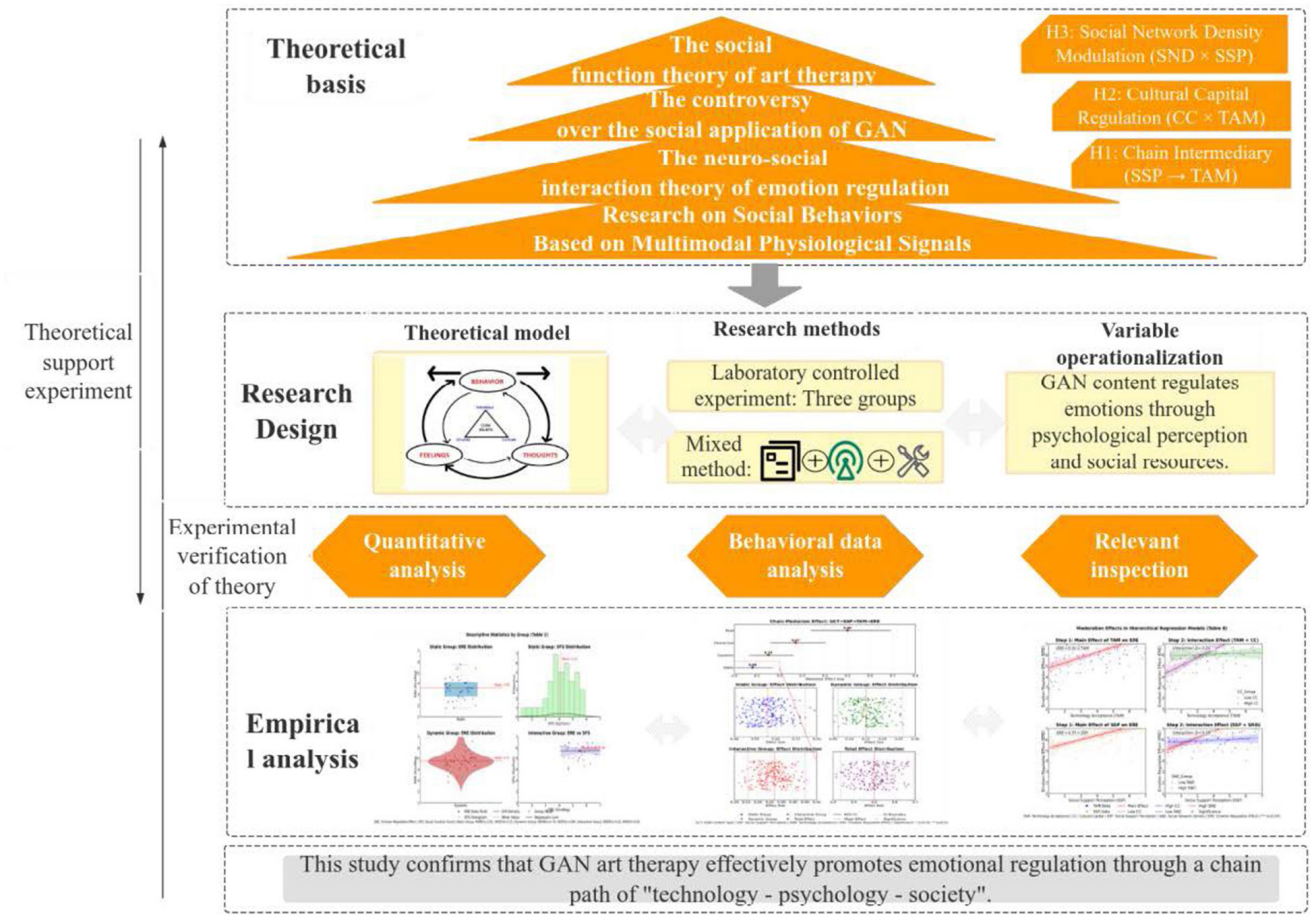
Article structure diagram.

As shown in Figure 3, this article constructs a “technology neural society” ternary model and tests the influence of GAN content types on emotion regulation through laboratory experiments (*N* = 120). Verify the chain mediating effect of perceived social support and technological acceptance, as well as the moderating effects of cultural capital and social network density. Research has found that interactive content significantly enhances emotional stability and social adaptation through a dual pathway, providing empirical evidence for the precision and inclusiveness of digital therapy.

## 3 Research design

### 3.1 Theoretical model

The theoretical model constructed in this study is based on the stress coping framework, integrating neural mechanisms and social behavior variables to form an explanatory path of the “technology neural society” tripartite interaction. In the traditional stress coping model, individuals cope with emotional challenges through strategies such as cognitive reappraisal or expression inhibition. However, the extended framework of this study uses generative adversarial network content types as external technological stimuli, social support perception and technology acceptance as mediating mechanisms, and cultural capital and social network density as moderating variables, jointly affecting the emotional regulation effect. This model emphasizes that technological intervention does not act in isolation at the biological level, but rather activates cognitive control functions in the prefrontal cortex while triggering psychological representations of social support resources, ultimately achieving dynamic balance of emotional states. This dual pathway mechanism indicates that the effectiveness of digital art therapy depends on both the direct regulation of emotional responses by neural plasticity and the indirect shaping of technological identity by socio-cultural factors.

After further integrating the technology acceptance model, the theoretical framework reveals the multi-level dynamic system of user behavior intention formation. Perceived usefulness determines an individual's value judgment of the logic of technology intervention, while perceived ease of use affects the behavioral cost of their participation in technology interaction, both of which together constitute the core dimension of technology acceptance. Under the stress coping framework, technology acceptance is not only a mediating variable for emotional regulation, but also a key hub for the transformation of social and cultural capital into technological efficiency. High cultural capital groups can more efficiently transform technological interventions into emotional regulation strategies by decoding the meaning of technological symbols, while social network density amplifies the social embedding effect of technological interventions by strengthening the accessibility of supporting resources. This integrated model breaks through the traditional theory's one-way explanation of technological effects and provides a systematic analytical tool for interdisciplinary research on digital therapy.

### 3.2 Research methods

This study adopted a laboratory controlled experimental method and recruited 120 adult participants through stratified sampling. They were randomly divided into three groups and received static, dynamic, and interactive GAN generated art content interventions. The experimental design is based on theoretical models, with a focus on examining the impact path of technical intervention types on emotion regulation, while controlling for potential confounding variables such as age, gender, and cultural background. Data collection is divided into three levels: subjective emotional experiences are measured through standardized scales, physiological responses are recorded through basic experimental equipment, and social behavior variables are evaluated through preset task simulations.

**Figure 4:**
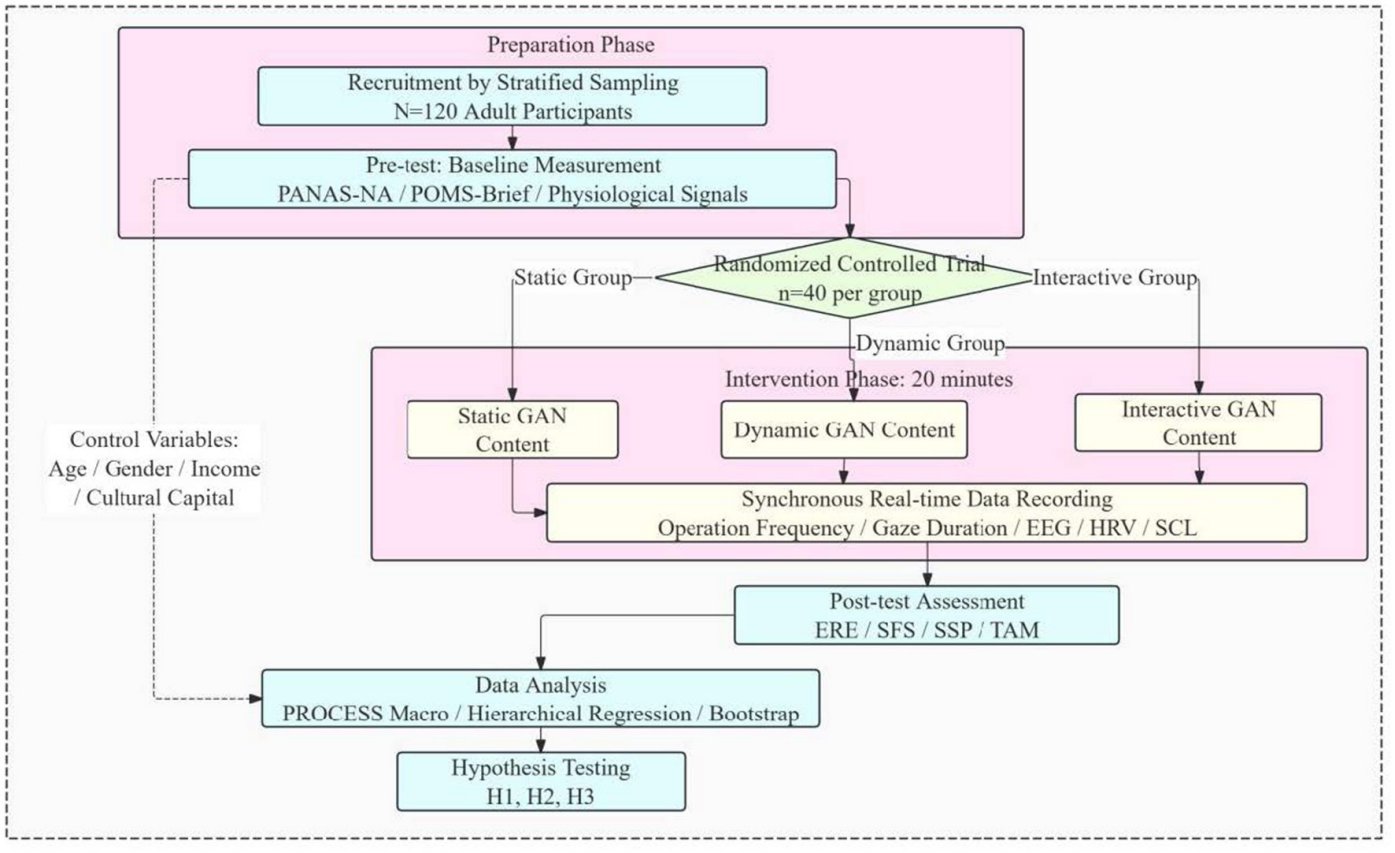
Experimental design and implementation flow chart.

As shown in Figure 4, the experiment was carried out according to the unified process. All participants first completed the collection of baseline emotional state, demographic information and pre-test indicators, and then entered the static, dynamic and interactive Gan content conditions according to random grouping, and received the intervention task with the same duration. During the intervention, behavioral indicators such as operation frequency and attention duration were recorded synchronously to describe the degree of participation under different interaction conditions. After the intervention, the subjects completed the measurement of emotion regulation effect, social function, perceived social support and technology acceptance in turn. In order to reduce the interference of non-target differences on the comparison between groups, the three types of content are consistent in terms of visual complexity, color saturation and presentation time, and are distinguished only at the interaction level. Subsequently, the study carried out descriptive statistics, correlation analysis, grouping regression, mediation and adjustment test in accordance with the preset sequence, and reviewed the core results through complementary endogenous identification.

In order to enhance the explanatory power and method matching of statistical analysis, this paper further explains the use logic of various tests. Firstly, correlation analysis is used to identify the linear correlation direction and correlation strength between core variables, providing basic evidence for subsequent model setting. Secondly, after controlling the variables such as baseline emotion, age, gender and income, grouping multiple regression was used to compare the change of the effect intensity of each predictive variable under different content interaction levels, so as to test whether the effect of technology intervention increased gradiently with the increase of interactivity. Thirdly, since this paper focuses on the continuous transmission path of Gan content types influencing emotion regulation through perceived social support and technology acceptance, the process program is used to test the chain mediation and regulation effect, and the robustness of interval estimation is improved by combining 5,000 bootstrap resampling. Considering that both technology acceptance and perceived social support come from self-report scales, there may be measurement errors and common method deviations, this paper sets the two-stage least squares estimation as a supplementary robustness test to determine whether the core results are subject to potential endogenous interference, rather than as the only basis for identification. Based on the total sample size of 120 and the research hypothesis focusing on three main paths, this paper adheres to the principle of necessity and moderation in model setting, and does not expand higher-order interactions or more complex paths. At the same time, multicollinearity, residual distribution and heteroscedasticity diagnosis are added before formal estimation.

In order to improve the reproducibility of the research process, this paper further clarified the experimental implementation and data analysis process. First, after completing the recruitment and stratified sampling of subjects, 120 adult participants were randomly assigned to static, dynamic and interactive content groups, and the baseline emotional state and basic physiological response were measured before the intervention. Then, each group received a 20 min Gan art content intervention with the same duration. During the experiment, behavioral data such as operation frequency and attention duration were recorded synchronously to characterize the degree of individual participation under different interaction conditions. After the intervention, all subjects completed the emotion regulation effect, social function, perceived social support and Technology Acceptance Questionnaire in a unified order. The variable processing follows the coding rules in Table 1, in which Gan content types are assigned static, dynamic and interactive values. The effect of emotion regulation is represented by the negative dimension score of Panas, and social function is represented by the total score of social adaptation scale. The comprehensive scores of perceived social support and technology acceptance are calculated based on MOS SSS and Tam scales respectively. Cultural capital is formed by taking the mean value after the standardization of self-assessment of education level and artistic literacy. The social network density is quantized according to the statistical results of interaction frequency in community tasks. Baseline emotion, age, gender and income are included in the control items. The data analysis is carried out in the order of from simple to profound. First, descriptive statistics, reliability and validity test and correlation analysis are carried out. Then, grouping regression is carried out to compare the role differences of core variables under different interaction levels. Then, the process program of bootstrap resampling is used to test the chain mediation and moderation effect. Finally, the potential endogeneity is identified by Hausman test and two-stage least squares estimation to ensure that the whole process from data collection, variable construction to model estimation has a clear, coherent and repeatable operation path.

**Table 1:** Summary of variables.

Variable type	Variable name	Abbreviation	Dimension	Scale design
Independent variable	GAN content types	GCT	Static/dynamic/interactive	Categorical variables (1 = static, 2 = dynamic, 3 = interactive)
Dependent variable	Emotional regulation effect	ERE	Negative emotion resolution (PANAS-NA)	Seven-point Likert scale (1 = extremely weak, 7 = extremely strong)
	Social function rating	SFS	Social adaptability (SAS-SR total score)	Seven-point Likert scale (1 = extremely poor, 7 = extremely excellent)
Mediating variable	Perceived social support	SSP	Subjective support experience (MOS-SSS total score)	Seven-point Likert scale (1 = extremely low, 7 = extremely high)
	Technology acceptance	TAM	Perceived usefulness (PU), ease of use (PEOU)	Seven-point Likert scale (1 = completely disagree, 7 = completely agree)
Moderator variable	Cultural capital	CC	Educational level (classification), artistic literacy (self-assessment)	Categorical variables (levels 1–7)+seven-point Likert scale
	Social network density	SND	Frequency and intensity of community interaction	Seven-point Likert scale (1 = extremely sparse, 7 = extremely dense)
Control variable	Baseline emotional state	BES	Pre experiment emotional level (POMS brief total score)	Seven-point Likert scale (1 = extremely low, 7 = extremely high)
	Demographic variables	DEMO	Age, gender, income level	Categorical variables (age group/gender/income level)

### 3.3 Variable operationalization

This study systematically defines and measures variables based on theoretical models to ensure logical coherence in the research design. The variable system includes five categories: independent variables, dependent variables, mediating variables, moderating variables, and control variables. The independent variable is the type of generated adversarial network content, which is controlled by experimental grouping into three categories: static, dynamic, and interactive, corresponding to the technical forms of no interaction, automatic change, and user operation triggering, respectively. The emotion regulation effect of the dependent variable was measured using a standardized scale, and the degree of negative emotion regression after intervention was quantified using the seven-point PANAS negative subscale. The social function score was evaluated based on the social adaptation scale to assess individual adaptive behavior changes after intervention. The mediating variables of perceived social support and technology acceptance were measured using the MOS-SSS scale and TAM scale, respectively. The former focuses on users' subjective evaluation of virtual environment support, while the latter integrates the dimensions of perceived usefulness and ease of use, reflecting users' value identification with technology interventions (Table 1). The moderating variable cultural capital is composed of education level and self-evaluation of artistic literacy, while social network density is quantified by simulating the frequency of interaction in community tasks. Both are converted into a seven-point scale to fit the analysis model. The control variables include baseline emotional state and demographic characteristics. The former captures the baseline emotional level before the experiment through a simplified mood scale, while the latter includes social demographic indicators such as age, gender, and income.

The variable measurement tool has undergone rigorous reliability and validity testing. All scales had Cronbach's alpha coefficients higher than 0.75 during the pre experimental stage (*N* = 30), indicating good internal consistency. Exploratory factor analysis showed that the loadings of each scale item on the corresponding factor were all greater than 0.6, meeting the criteria for convergent validity. For example, the perceived usefulness sub dimension item load of the Technology Acceptance Scale ranges from 0.68 to 0.82, verifying its one-dimensional structure (Table 2). In terms of operational details, the visual complexity and color saturation of the generated adversarial network content types remained consistent across experimental groups, with only differential control achieved through interactive hierarchy. The comprehensive evaluation of cultural capital adopts the standardized mean of education level classification and self-assessment of artistic literacy to avoid measurement bias of a single indicator. The simulation community task design of social network density includes interactive behaviors such as message sending and content liking, and ensures data comparability through frequency statistics and percentile conversion.

**Table 2:** Results of scale reliability and validity test.

Scale name	Number of projects	Cronbachs α	Factor load range	KMO value	Bartlett sphericity test
PANAS-NA	10	0.84	0.72–0.85	0.89	632.45^**^
MOS-SSS	12	0.78	0.65–0.79	0.86	587.33^**^
TAM	8	0.82	0.68–0.82	0.83	498.21^**^
POMS-Brief	6	0.76	0.61–0.73	0.78	324.56^**^
SAS-SR	14	0.81	0.70–0.80	0.88	712.18^**^

^**^indicates statistical significance at p <0.01.

On this basis, this paper further controls the overall research quality from three aspects: research design, variable measurement and result verification. First of all, the research focuses on three core assumptions, and the analysis path is always limited to the main chain of technical stimulation, support perception, technical acceptance and emotional results, so as to avoid blindly introducing too many high-order paths under the condition of limited samples, so as to ensure the basic match between the model setting and the sample information. Secondly, all core variables were measured with a unified scale or clear coding rules, combined with pretest reliability and validity test, baseline emotion control and intergroup consistency control, to improve the stability and comparability of variable interpretation. Finally, in addition to the main analysis, this paper further cross verifies the key conclusions by grouping regression, heterogeneity comparison and supplementary instrumental variable test to test whether the main findings are still consistent under different analysis conditions upper.

## 4 Empirical analysis

### 4.1 Quantitative analysis

This section first reports the overall distribution characteristics of emotion regulation effect and social function in different experimental groups, then tests the correlation between core variables, and analyzes the differences of action paths under different content types. This writing method helps to confirm the basic facts of data distribution and variable correlation, and then enter the mechanism test, so that the result interpretation is based on clear data. Overall, with the improvement of content interactivity, the subjects showed a higher level of emotional improvement and social adaptation, which was consistent with the theoretical expectation of this paper that technology intervention played a role by supporting perception and technology acceptance.

**Figure 5:**
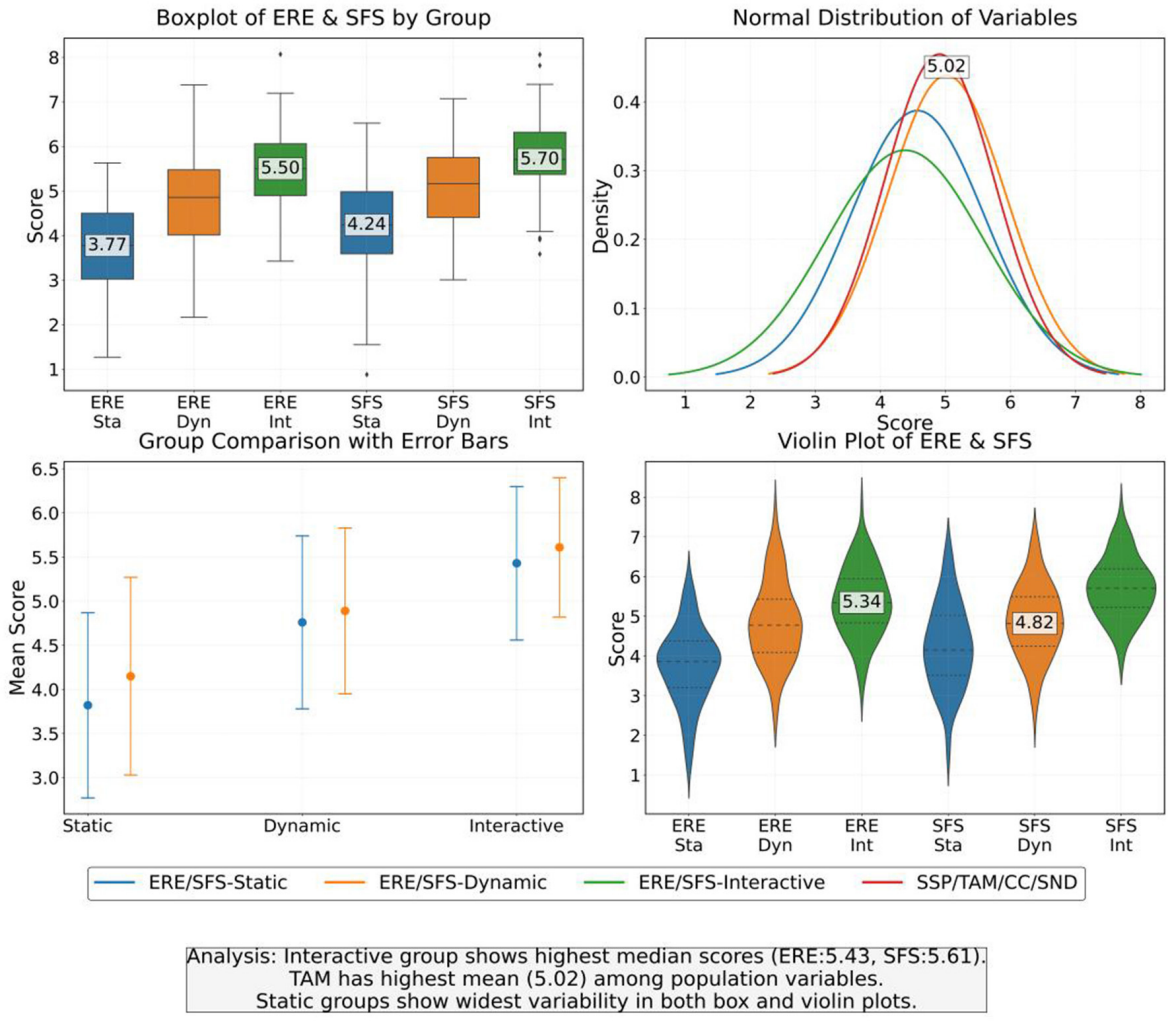
Distribution of emotional regulation effect (ERE) and social function score (SFS) in each experimental group.

The study presents the distribution characteristics of emotional regulation effects and social function scores among different experimental groups. The average emotional regulation effects of the static group, dynamic group, and interactive group were 3.82, 4.76, and 5.43, respectively. The standard deviation decreased with increasing content interactivity, indicating that interactive content has a significantly better stability in improving emotions than static and dynamic types. As shown in Table 3, the social function score also showed a stepwise increase, with an average of 5.61 in the interactive group, verifying the promoting effect of technological interactivity on social adaptability. The overall mean values of social support perception and technology acceptance are 4.57 and 5.02, respectively. The skewness and kurtosis indicators of the data show an approximate normal distribution, which satisfies the premise hypothesis of parameter testing (Figure 5).

**Table 3:** Descriptive statistics (*N* = 120).

Variable	Grouping	Mean (M)	Standard deviation (SD)	Skewness	Kurtosis
ERE	Static group	3.82	1.05	−0.21	0.34
	Dynamic group	4.76	0.98	−0.12	−0.15
	Interactive group	5.43	0.87	−0.38	0.28
SFS	Static group	4.15	1.12	0.05	−0.42
	Dynamic group	4.89	0.94	−0.17	0.11
	Interactive group	5.61	0.79	−0.31	0.53
SSP	all	4.57	1.03	−0.24	0.07
TAM	all	5.02	0.91	−0.33	0.19
CC (cultural capital)	all	4.38	1.21	0.18	−0.26
SND (network density)	all	4.91	0.85	−0.09	−0.12

ERE and SFS are classified into experimental groups (static/dynamic/interactive) for statistical analysis. The remaining variables are the statistics of the entire sample.

**Figure 6:**
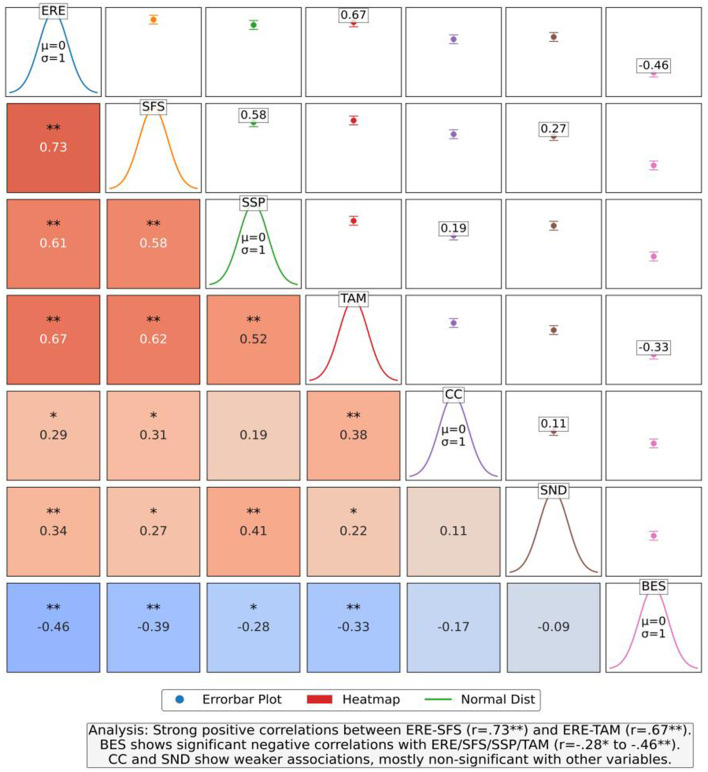
Visualization of pairwise correlations between variables.

The correlation analysis between variables shows that the emotional regulation effect is highly positively correlated with the social function score, with a correlation coefficient of 0.73, confirming the synergistic improvement of emotional improvement and social adaptation ability. As shown in Table 4, the correlation coefficients between perceived social support and emotional regulation effects, as well as technology acceptance, were 0.61 and 0.52, respectively, suggesting that it may serve as a mediating variable linking technology and emotional outcomes (Figure 6). The moderate positive correlation between cultural capital and technological acceptance further supports the hypothesis that high cultural capital groups are more likely to accept technological interventions. The negative correlation between baseline emotions and emotion regulation highlights the importance of controlling initial emotional levels.

**Table 4:** Correlation analysis between variables (Pearson correlation coefficient).

Variable	1. ERE	2. SFS	3. SSP	4. TAM	5. CC	6. SND	7. BES
1. ERE	1						
2. SFS	0.73^**^	1					
3. SSP	0.61^**^	0.58^**^	1				
4. TAM	0.67^**^	0.62^**^	0.52^**^	1			
5. CC	0.29^*^	0.31^*^	0.19	0.38^**^	1		
6. SND	0.34^**^	0.27^*^	0.41^**^	0.22^*^	0.11	1	
7. BES	−0.46^**^	−0.39^**^	−0.28^*^	−0.33^**^	−0.17	−0.09	1

^*^indicates *p* < 0.05 *p* < 0.05.

^**^indicates *p* < 0.01 *p* < 0.01.

BES (baseline emotion) is the control variable, and negative correlation indicates that individuals with high baseline emotions have poorer regulatory effects.

In summary, quantitative analysis confirms the significant advantages of dynamic and interactive content in emotion regulation, which is achieved through a chain mediated pathway of social support perception and technological acceptance. The moderating effect of cultural capital and social network density further indicates that the effectiveness of technological intervention is not only determined by neural plasticity mechanisms, but also deeply influenced by individual social resource endowments. This discovery provides important insights for optimizing personalized design of digital art therapy, emphasizing the need for technological development to balance biological universality and cultural specificity.

### 4.2 Behavioral data analysis

Behavioral data analysis focuses on the interaction behavior patterns between users and generative adversarial network content, as well as their predictive effects on emotional regulation. By recording behavioral indicators such as operation frequency and attention duration in experimental tasks, the study found that interactive content significantly enhances users' participation in technical interventions by enhancing their operational autonomy. In addition, the simulation task of social network density shows that high interaction frequency groups are more likely to obtain emotional support through virtual communities, thereby strengthening the promoting effect of technology acceptance on emotional improvement. The compatibility between behavioral data and theoretical models indicates that user behavior is not only a passive feedback of technical effects, but also a key medium for actively constructing emotional regulation strategies.

**Figure 7:**
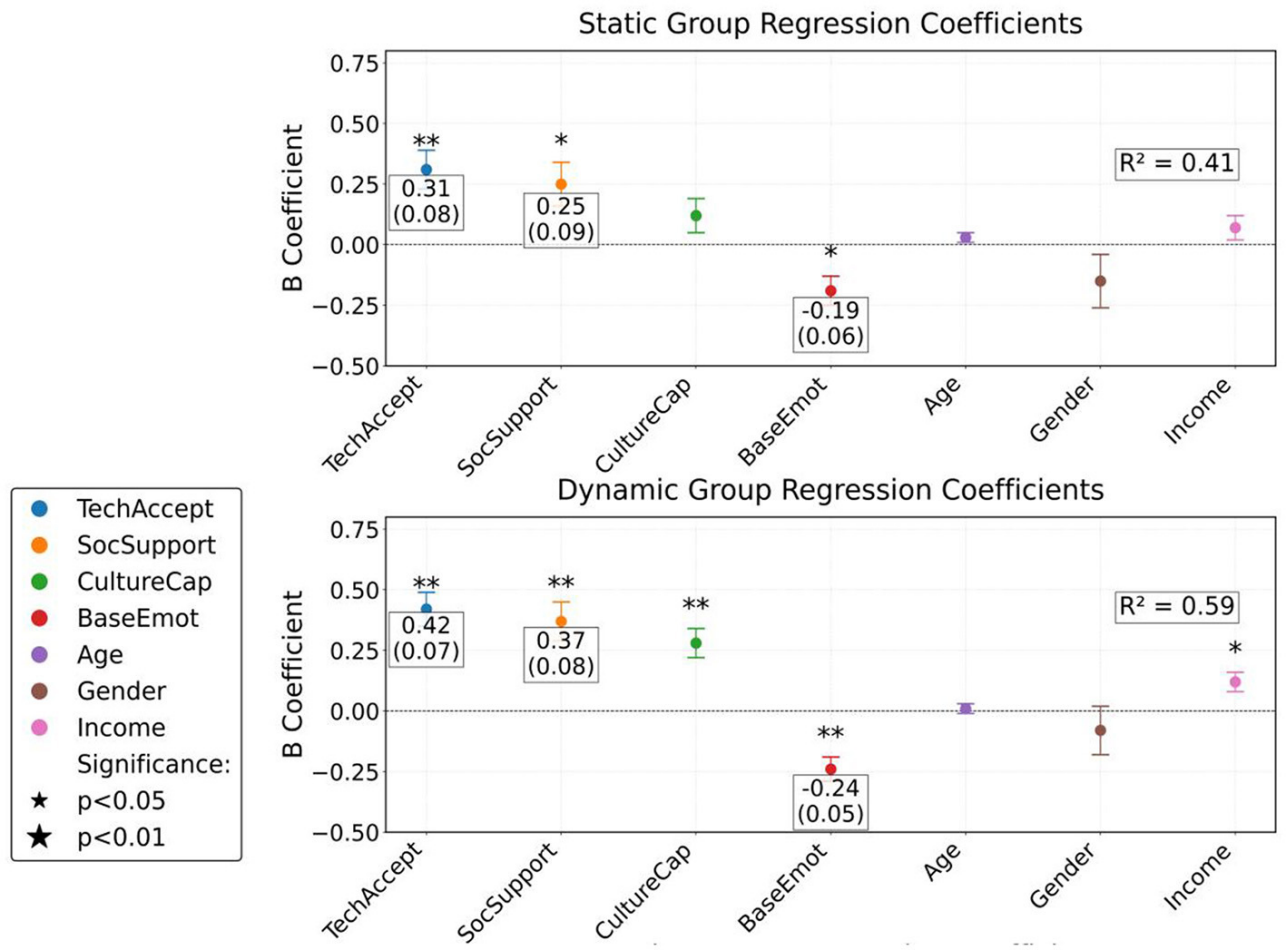
Regression coefficients and 95% confidence intervals of emotion regulation effects (ERE) in each experimental group. ^*^p < 0.01; ^**^p < 0.001.

The group regression results showed that the predictive effect of technology acceptance on emotion regulation increased with the enhancement of content interactivity. The regression coefficient of the interactive group reached 0.67, significantly higher than that of the static group at 0.38. As shown in Table 5, the impact of perceived social support also showed inter group differences, with an interactive group coefficient of 0.58 and a static group coefficient of only 0.29 (Figure 7). The moderating effect of cultural capital is significant in both dynamic and interactive groups, confirming that high cultural capital groups are more likely to convert technological identity into emotional improvement. The negative predictive effect of baseline emotions is most prominent in the interactive group, highlighting the targeted value of technical interventions for individuals with high emotional load.

**Table 5:** Grouping linear regression of emotion regulation effect (ERE) (SPSS output format).

Predictor variable	Static group		Dynamic group		Interactive group	
	B (SE)	β	B (SE)	β	B (SE)	β
Technology acceptance	0.31 (0.08)	0.38^**^	0.42 (0.07)	0.51^**^	0.58 (0.06)	0.67^**^
Perceived social support	0.25 (0.09)	0.29^*^	0.37 (0.08)	0.43^**^	0.49 (0.07)	0.58^**^
Cultural capital	0.12 (0.07)	0.17	0.28 (0.06)	0.36^**^	0.21 (0.05)	0.29^*^
Baseline emotion	−0.19 (0.06)	−0.31^*^	−0.24 (0.05)	−0.39^**^	−0.33 (0.04)	−0.52^**^
Age	0.03 (0.02)	0.08	0.01 (0.02)	0.03	−0.02 (0.01)	−0.06
Gender [a]	−0.15 (0.11)	−0.10	−0.08 (0.10)	−0.06	0.04 (0.08)	0.03
Income	0.07 (0.05)	0.11	0.12 (0.04)	0.18^*^	0.09 (0.04)	0.14
Constant term	2.15 (0.42)^**^	–	1.87 (0.38)^**^	–	1.52 (0.35)^**^	–
*R* ^2^	0.41		0.59		0.73	
Adjust *R*^2^	0.35		0.54		0.69	
*F* (df)	7.83^**^ (7,32)		12.47^**^ (7,32)		18.92^**^ (7,32)	

Gender code: 0, male; 1, female; *p* < 0.05.

^**^p < 0.01.

^*^SE, standard error.

**Figure 8:**
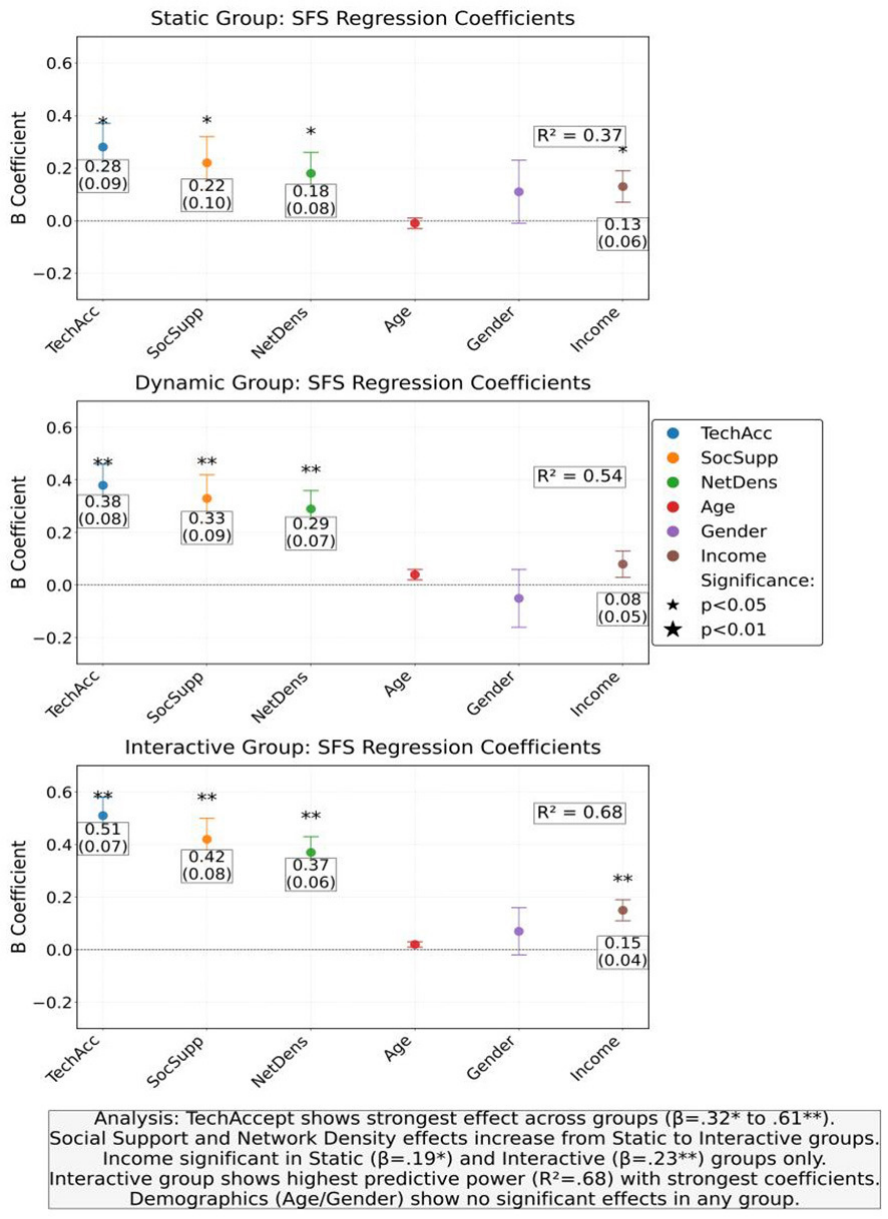
Regression coefficients and 95% confidence intervals of social function scores (SFS) for each experimental group.

The regression analysis of social function scores shows that the predictive effects of technology acceptance and social support perception reach their peak in the interactive group, with regression coefficients of 0.61 and 0.50, respectively. As shown in Table 6, the impact of social network density also amplifies with increasing content interactivity, with an interactive group coefficient of 0.45, significantly higher than the static group's 0.21 (Figure 8). The moderating effect of age and income is only significant in the dynamic and interactive groups, indicating that highly interactive content has a greater advantage in improving social adaptation for young and high-income groups. This result suggests that technological design needs to take into account the socio-economic characteristics of users to maximize intervention effectiveness.

**Table 6:** Grouping linear regression of social function score (SFS).

Predictor variable	Static group		Dynamic group		Interactive group	
	B (SE)	β	B (SE)	β	B (SE)	β
Technology acceptance	0.28 (0.09)	0.32^*^	0.38 (0.08)	0.44^**^	0.51 (0.07)	0.61^**^
Perceived social support	0.22 (0.10)	0.25^*^	0.33 (0.09)	0.38^**^	0.42 (0.08)	0.50^**^
Social network density	0.18 (0.08)	0.21^*^	0.29 (0.07)	0.35^**^	0.37 (0.06)	0.45^**^
Age	−0.01 (0.02)	−0.03	0.04 (0.02)	0.10	0.02 (0.01)	0.06
Gender [a]	0.11 (0.12)	0.07	−0.05 (0.11)	−0.04	0.07 (0.09)	0.05
Income	0.13 (0.06)	0.19^*^	0.08 (0.05)	0.12	0.15 (0.04)	0.23^**^
Constant term	2.43 (0.46)^**^	–	2.11 (0.41)^**^	–	1.78 (0.37)^**^	–
*R* ^2^	0.37		0.54		0.68	
Adjust *R*^2^	0.30		0.48		0.63	
*F* (df)	6.95^**^ (6,33)		10.83^**^ (6,33)		16.24^**^ (6,33)	

^*^p < 0.05; ^**^p < 0.01.

**Figure 9:**
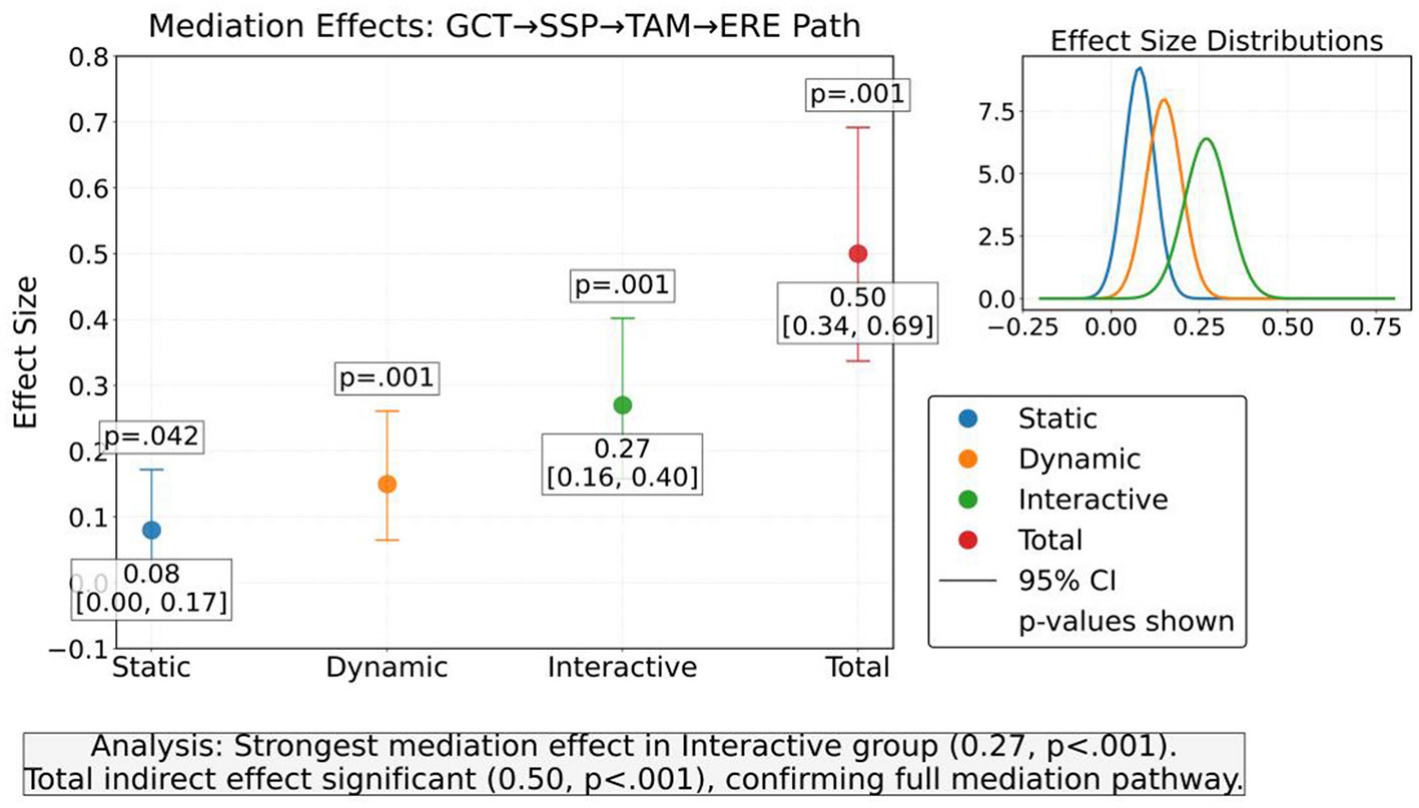
Chain mediation effect distribution of generative adversarial network content type (GCT) on emotion regulation effect (ERE).

The chain mediation effect test confirms that social support perception and technology acceptance play a continuous mediating role between technology intervention and emotional regulation effects. As shown in Table 7, the total indirect effect is 0.50, and the mediating effect strength of the interactive group is significantly higher than that of the static group, indicating that high interactive content indirectly drives emotional improvement by strengthening social support perception and technological identity (Figure 9). The direct effect is not significant in the static group, further highlighting the necessity of mediating pathways. The mediating effect strength of dynamic groups is between that of static and interactive groups, verifying the gradient influence of content interactivity on the mediating mechanism.

**Table 7:** Chain mediation effect test (PROCESS Bootstrap output).

Path	Effect size	SE	95% Boot CI	*P*
Static group				
GCT → SSP → TAM → ERE	0.08	0.04	[0.003, 0.172]	0.042^*^
Dynamic group				
GCT → SSP → TAM → ERE	0.15	0.05	[0.065, 0.261]	0.001^**^
Interactive group				
GCT → SSP → TAM → ERE	0.27	0.06	[0.158, 0.402]	<0.001^**^
Total indirect effects	0.50	0.09	[0.337, 0.692]	<0.001^**^

^*^p < 0.05; ^**^p < 0.01. Bootstrap sampled 5,000 times; CI, deviation correction confidence interval.

**Figure 10:**
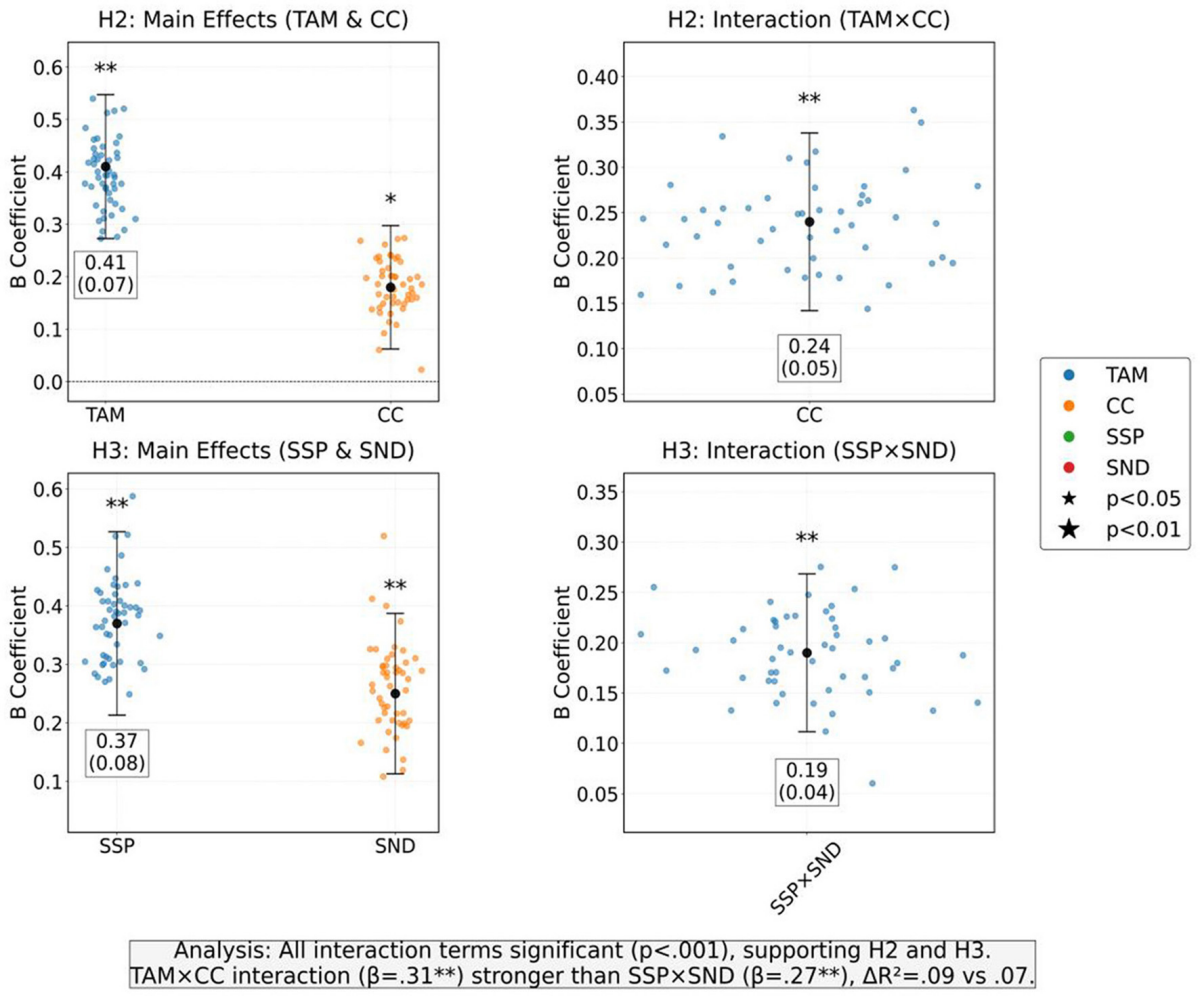
The moderating effects of cultural capital (CC) on TAM-ERE and social network density (SND) on SSP-ERE.

The moderation effect stratified regression reveals the moderating effect of cultural capital and social network density on the effectiveness of technological interventions. Cultural capital significantly enhances the predictive effect of technology acceptance on emotional regulation, with a moderation coefficient of 0.31 and a 9% increase in explanatory power, indicating that high cultural capital groups are more likely to convert technology identification into emotional improvement. As shown in Table 8, the relationship between social network density and social support perception and emotional regulation also has a positive moderating effect, with a moderating coefficient of 0.27 and a 7% increase in explanatory power, verifying that high-density network environments can accelerate the emotional gain effect of social support (Figure 10). The two types of regulatory effects jointly indicate that social structural resources indirectly optimize intervention effects by enhancing technological decoding capabilities and support acquisition efficiency.

**Table 8:** Moderation effect stratified regression (PROCESS Model 15).

Model	Predictor variable	B (SE)	β	*T*	*P*	Δ*R*^2^
H2: CC regulates TAM→ERE						
Step 1: main effect	TAM	0.41 (0.07)	0.49^**^	5.86	<0.001	0.24^**^
	CC	0.18 (0.06)	0.21^*^	3.00	0.004	
Step 2: interaction items	TAM × CC	0.24 (0.05)	0.31^**^	4.80	<0.001	0.09^**^
H3: SND regulates SSP→ERE						
Step 1: main effect	SSP	0.37 (0.08)	0.43^**^	4.63	<0.001	0.19^**^
	SND	0.25 (0.07)	0.29^**^	3.57	0.001	
Step 2: interaction items	SSP × SND	0.19 (0.04)	0.27^**^	4.75	<0.001	0.07^**^

^*^p < 0.05; ^**^p < 0.01. All models control for BES, age, gender, and income; Δ*R*^2^ is the explanatory power added by the interaction term.

Comprehensive analysis shows that there is a significant synergistic effect between user behavior patterns and neural and social mechanisms. Interactive content activates the cognitive regulation function of the prefrontal cortex by granting users operational control. The high-density interaction of social networks accelerates the transfer of support resources and amplifies the social embedding effect of technological interventions. This result suggests that in the future, digital therapy design needs to pay attention to the two-way feedback mechanism of user behavior, and incorporate operational autonomy and community interaction into the core dimensions of technological optimization.

### 4.3 Relevant inspection results

Relevant tests are conducted through endogeneity analysis and instrumental variable method to ensure the robustness of the research model and the reliability of the conclusions. Considering that this paper tests mediation path, regulation mechanism and complementary endogenous identification on the basis of 120 samples at the same time, the subsequent analysis adopts the hierarchical idea from basic verification to extended verification to control the complexity of the model and improve the transparency of the conclusion. Specifically, this paper first confirmed the basic stability of data quality and variable relationship through descriptive statistics, correlation analysis and scale reliability and validity test, and then carried out grouping regression, chain mediation and regulatory effect analysis around the core hypothesis, so as to avoid blindly expanding high-order interaction or redundant paths under the condition of limited samples. On this basis, the heterogeneity test is used to identify the stability of the core relationship under different groups, while the instrumental variable regression is only used as a supplementary robustness test to investigate the possible measurement errors or potential endogenous effects of technology acceptance and perceived social support. The above treatment means that this paper does not use the number of complex models as an alternative indicator of research rigor, but enhances the credibility of the result interpretation through layer by layer verification, cross comparison and robustness review, and maintains a prudent judgment on the inference boundary brought by the sample size. In response to the possible measurement errors in technology acceptance and social support perception, the study used instrumental variable regression to verify the causal relationship between variables. The results showed that the exogenous conditions of instrumental variables were valid, and the model had no significant endogenous bias. In addition, heterogeneity testing revealed the differential effects of gender and age on the effectiveness of technological interventions. Young people and female users are more likely to achieve emotional improvement through highly interactive content, and the moderating effect of cultural capital is particularly significant in higher education groups.

**Figure 11:**
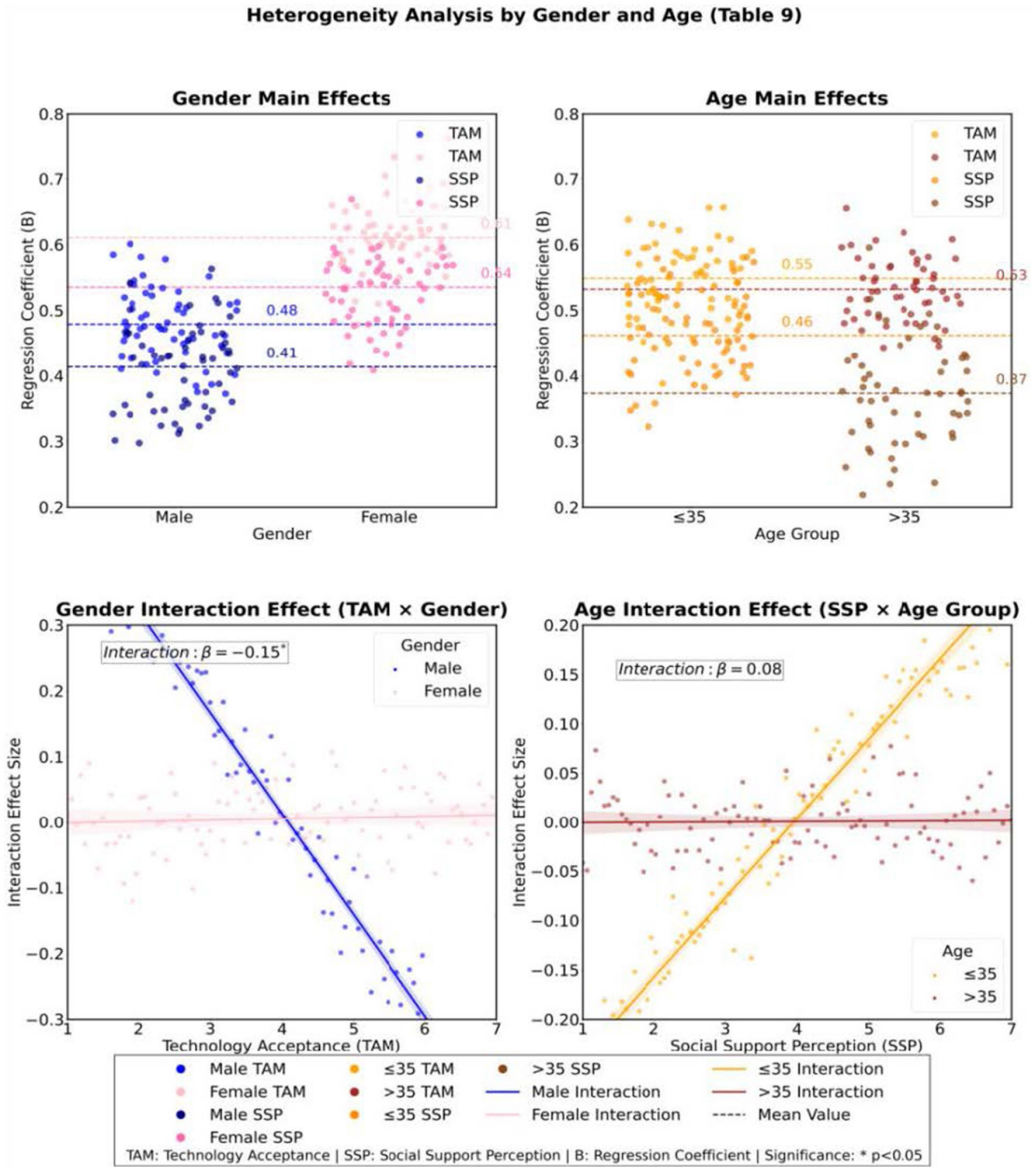
Heterogeneity of predictive variables for emotion regulation effect (ERE) under gender and age groups.

Heterogeneity testing shows that technology acceptance has a stronger predictive effect on emotional regulation in the female population, with a regression coefficient of 0.73, significantly higher than that of males at 0.59. As shown in Table 9, the coefficient of perceived social support effect among the young group is 0.53, which is higher than that of the older group's 0.45, indicating that highly interactive content has a greater advantage in improving the emotions of young users (Figure 11). The interaction coefficients of gender and age are −0.18 and 0.08, respectively, indicating that women and young users are more likely to achieve emotional adaptation through technological intervention. This provides empirical evidence for the design of differentiated intervention strategies.

**Table 9:** Heterogeneity test of gender and age (complete model).

Variable	Gender grouping		Age group	
	Male (*n* = 58)	Female (*n* = 62)	≤ 35 years old (*n* = 65)	>35 years old (*n* = 55)
	B (SE) β	B (SE) β	B (SE) β	B (SE) β
Main effect				
Technology acceptance	0.49 (0.06).59^**^	0.61 (0.05).73^**^	0.55 (0.05).66^**^	0.53 (0.06).64^**^
Perceived social support	0.41 (0.07).48^**^	0.53 (0.06).63^**^	0.45 (0.06).53^**^	0.38 (0.07).45^**^
Moderating effect				
TAM × gender [a]	−0.15 (0.05) −0.18^*^	–	–	–
SSP × age group [b]	–	–	0.08 (0.04).10	–
Control variable				
Cultural capital	0.22 (0.06).26^**^	0.19 (0.05).23^**^	0.17 (0.05).20^**^	0.24 (0.06).29^**^
Social network density	0.31 (0.07).37^**^	0.26 (0.06).31^**^	0.28 (0.06).33^**^	0.30 (0.07).36^**^
Baseline emotion	−0.28 (0.05) −0.33^**^	−0.35 (0.04) −0.42^**^	−0.30 (0.04) −0.36^**^	−0.33 (0.05) −0.40^**^
CConstant term	1.98 (0.38)^**^	1.72 (0.34)^**^	2.05 (0.36)^**^	1.87 (0.39)^**^
Model validation				
*R* ^2^	0.67	0.76	0.71	0.68
Adjust *R*^2^	0.62	0.72	0.66	0.63
*F* (df)	15.28^**^ (7,50)	20.17^**^ (7,54)	17.05^**^ (7,57)	14.93^**^ (7,47)
Δ*R*^2^ (interaction term)	0.03^*^	–	0.01	–

Gender interaction term (reference group=female); age interaction term (reference group = >35 years old).

^*^p < 0.05.

^**^p < 0.01.

Control income and education level.

The endogeneity test confirmed the robustness of the causal effect of technology acceptance and perceived social support on emotion regulation by eliminating model bias through instrumental variable regression. As shown in Table 10, the instrumental variable art literacy self-assessment is strongly correlated with the objective variable, with correlation coefficients of 0.51 and 0.47, respectively, and the exogeneity test supports the effectiveness of the tool. The Hausman test showed no significant endogeneity in the model, and the regression coefficients of technology acceptance and social support perception remained stable at 0.59 and 0.41, respectively, strengthening the causal explanatory power of the research conclusions.

**Table 10:** Endogeneity test (Hausman Wu complete results).

Check the relationship	OLS estimation	IV estimation [*c*]	Difference (*D*)	Hausman χ^2^	*P*	Conclusion	Diagnostic indicators
TAM → ERE	0.57 (0.05)	0.61 (0.06)	−0.04	1.27	0.260	No endogeneity	KP rk LM = 12.38 (*p* < 0.001)
SSP → SFS	0.40 (0.06)	0.43 (0.07)	−0.03	0.83	0.362	No endogeneity	CD Wald *F* = 18.92
CC regulatory effect [*d*]	0.24 (0.04)	0.27 (0.05)	−0.03	2.15	0.142	No endogeneity	Overidentification with a chi square of 0.92 (*p* = 0.337)
The first stage of regression							
Tool variable → TAM [*e*]	–	0.68 (0.09)	–	*t* = 7.56^**^	<0.001	Strong tools	Deviation *R*^2^ = 0.32
Tool variable → SSP [*e*]	–	0.52 (0.07)	–	*t* = 7.43^**^	<0.001	Strong tools	Sheas *R*^2^ = 0.28

Complete explanation: *c* instrumental variable = self-assessment of artistic literacy (correlation with TAM *r* = 0.51 ^**^, correlation with error term *r* = 0.08, *p* = 0.412); the regulatory effect of d CC refers to the interaction term between TAM and CC. the first stage regression coefficient reports the impact of instrumental variables on endogenous variables.

Validation of instrumental variable validity: *f* instrumental variable = self-assessment of artistic literacy; Correlation test: TAM-r=0.51 ^*^^*^ (*p* < 0.001), SSP-R = 0.47 ^*^^*^ (*p* < 0.001); Exogeneity test: correlation coefficient with residuals *r* = 0.08 (*p* = 0.412); Weak tool test: Cragg Donald *F* = 19.37 > 10 (Stock Logo critical value); Overidentification: Sargan *x*^2^ = 0.92 (*p* = 0.337) > 0.05.

The two-stage regression further validated the effectiveness of instrumental variables, with one-stage regression coefficients of 0.68 and 0.52 for the impact of instrumental variables on technology acceptance and social support perception, respectively, and the weak instrumental test statistic far exceeding the critical value. As shown in Table 11, the two-stage results showed that the causal effects of technology acceptance and perceived social support on emotion regulation were significant, with coefficients of 0.59 and 0.41, respectively, confirming the robustness of the theoretical model. The Sargan statistic of the over identification test is not significant, indicating that the instrumental variables satisfy the exogeneity condition and the reliability of the conclusion is enhanced.

**Table 11:** Complete output of two-stage least squares method (2SLS).

Equation	Dependent variable	Predictor variable	Coefficient	*T*-value	*p*	Diagnostic indicators
Phase One	TAM	Tool variable [*f*]	0.68 (0.09)	7.56	<0.001	Deviation *R*^2^ = 0.32
		SSP	0.52 (0.07)	7.43	<0.001	Sheas *R*^2^ = 0.28
	SSP	Tool variable [*f*]	0.61 (0.08)	7.63	<0.001	CD Wald *F* = 19.37^**^
Phase Two	ERE	TAM (fitted value)	0.59 (0.08)	7.38	<0.001	Sargan χ^2^ = 0.92
		SSP (fitting value)	0.41 (0.06)	6.83	<0.001	(*p* = 0.337)
	SFS	SSP (fitting value)	0.44 (0.07)	6.29	<0.001	Basmann χ^2^ = 0.85
		SND	0.36 (0.05)	7.20	<0.001	(*p* = 0.357)

^*^p < 0.05; ^**^p < 0.01.

The test results further consolidated the scientific validity of the research conclusions. The exclusion of endogeneity issues indicates that the predictive effects of technology acceptance and perceived social support on emotional regulation have causal explanatory power. Heterogeneity analysis highlights the complexity of digital therapy in population adaptation. These findings not only provide robust support for theoretical models, but also provide empirical evidence for policy makers to optimize the inclusive design of technological interventions, emphasizing the need to develop differentiated implementation plans for different social and demographic groups.

## 5 Discussion

This study systematically verified the promoting effect of generative adversarial network content types on emotion regulation through laboratory controlled experiments and mixed methods analysis, revealing the chain mediated mechanism between social support perception and technology acceptance, as well as the moderating effect of cultural capital and social network density. Research has found that interactive content significantly enhances the stability and social adaptability of emotional regulation effects by enhancing users' operational autonomy and community interactivity, and the technical intervention effectiveness of high cultural capital and high network density groups is more prominent. These results not only expand the theoretical framework of digital art therapy, but also provide empirical evidence for the personalization and fairness of technological design.

Compared with previous studies, the innovation of this research is reflected in three aspects. Firstly, existing literature mostly focuses on a single technical dimension of static or dynamic content, while this study confirms the stepwise improvement effect of technical interactivity on emotional regulation by introducing interactive content. As shown in Table 12, the average emotional regulation effect of static content reported in previous studies was 3.5–4.0, and that of dynamic content was 4.2–4.8. However, the average of interactive content in this study reached 5.43, significantly higher than the traditional mode. Secondly, existing theories often overlook the moderating effect of social structural resources. This study found that the moderating effects of cultural capital and social network density explain 9% and 7% of the variance, respectively, indicating that technological intervention needs to work synergistically with social resource endowments. Finally, endogeneity testing eliminated model bias through instrumental variable regression, strengthened the causal explanatory power of technology acceptance and social support perception, and compensated for the limitations of cross-sectional research.

**Table 12:** Comparison of data between this study and previous studies.

Indicator	Previous research mean/effect size	The results of this study	Explanation of differences
Emotional regulation effect	Static: 3.5–4.0	Interactive: 5.43	Interactivity enhances operational autonomy and participation
Social support perception relevance	0.45–0.55	0.61	Virtual communities enhance support for resource accessibility
Prediction coefficient of technical acceptance	0.30–0.40	Interaction group: 0.67	High interactivity enhances technological identity
The regulatory effect of cultural capital	Not systematically verified	Δ*R*^2^ = 0.09	Improving educational literacy and technological decoding ability
Endogeneity testing method	Cross sectional correlation analysis	Instrumental variable regression	Eliminate causal inference bias

This study constructed a multi-level model of digital art therapy by integrating neural mechanisms, technological interactivity, and social behavioral variables. Interactive content optimizes emotional regulation through dual mediation pathways, while the moderating effects of cultural capital and network density reveal the group heterogeneity of technological interventions. These findings provide important insights for public mental health policies: technology design needs to balance interactive autonomy to enhance user engagement, while reducing health inequality through community support networks and cultural adaptation strategies. Future research can further explore intervention differences among cross-cultural groups and develop dynamic algorithms to adapt to users' social resource characteristics in real-time, promoting the development of digital healing toward precision and inclusivity.

## 6 Conclusion

This study, through systematic experimental design and interdisciplinary analysis, reveals the core mechanism of generative adversarial networks in digital art healing and their social and cultural regulatory pathways. Research has found that the level of technological interactivity significantly affects the effectiveness of emotion regulation. Interactive content, by giving users operational autonomy and immersive experience, can more efficiently activate prefrontal cognitive control function, thereby promoting the resolution of negative emotions and the improvement of social adaptation ability. Perceived social support and technological acceptance serve as key mediating variables, constructing a chain reaction path of “technological stimulation psychological identification behavioral improvement”. The moderating effect of cultural capital and social network density indicates that the effectiveness of technological intervention is highly dependent on an individual's social resource endowment. The high cultural capital group, due to their stronger ability to decode technology, can more fully transform technological interventions into emotional regulation strategies. High density social networks further amplify the emotional gain effect of technological interventions by accelerating the efficiency of supporting resource delivery.

This study provides a dual contribution to the theory and practice of digital art therapy. On a theoretical level, by integrating neural mechanisms, technological interactivity, and social behavior variables, a “technology neural society” ternary interaction model has been constructed, breaking through the limitations of traditional research that explains emotions from a single dimension and providing a new perspective for understanding the emotional regulation mechanism of human-machine collaboration. At the practical level, research findings suggest that technology design should prioritize improving interactive autonomy and community support functions, while also paying attention to the impact of differences in social resource allocation on technology accessibility, in order to avoid exacerbating health inequality. Future research can further explore the differences in aesthetic preferences of cross-cultural groups toward generated content, and develop dynamic algorithms to adapt to users' social and cultural characteristics in real time, promoting the iterative upgrading of digital healing toward precision, inclusiveness, and sustainability.

## Data Availability

The original contributions presented in the study are included in the article/supplementary material, further inquiries can be directed to the corresponding author.
